# Same Surgeon: Different Centre Equals Differing Lymph Node Harvest following Colorectal Cancer Resection

**DOI:** 10.1155/2011/406517

**Published:** 2011-03-17

**Authors:** M. D. Evans, S. Robinson, S. Badiani, A. Rees, J. D. Stamatakis, S. S. Karandikar, G. Langman

**Affiliations:** ^1^Department of Surgery, Singleton Hospital, Sketty, Swansea SA2 8QA, UK; ^2^Alexandra Hospital, Worcester Acute Hospital NHS Trust, Redditch B98 7UB, UK; ^3^Department of Surgery, Heart of England Foundation NHS Trust, Birmingham B9 5SS, UK; ^4^Princess of Wales Hospital, Bridgend CF31 1RQ, UK; ^5^Department of Pathology, Heart of England Foundation NHS Trust, Birmingham B9 5SS, UK

## Abstract

*Introduction*. The aim of this study was to examine the effect of surgeon relocation on lymph node (LN) retrieval in colorectal cancer (CRC) resection. *Methods*. The study population was 213 consecutive patients undergoing CRC resection by a single surgeon, at two units: unit one 110 operations (2002–2005) and unit two 103 (2005–2009). LN yields and case mix were compared. *Results*. Median LN harvests were significantly different between the two centres: unit 1: 13 nodes/patient and unit 2: 22 nodes/patient (*P* < .001). In unit one 42% of cases were LN positive and in unit two 48% (*P* = .398). There was no difference in case mix. Multivariate analysis identified unit (*P* < .001) and pathologist (*P* = .007) as independent predictors of harvest. *Conclusions*. A surgeon moving units can experience significantly different LN yield following CRC resection. Both units comply with national standards, but the “surgeon's results” at the two units appear to be pathologist dependent. This has implications for nodal harvest as a surrogate marker of surgical quality.

## 1. Introduction

The identification of lymph node (LN) metastases following colorectal cancer (CRC) resection is one of the critical discriminators that influence the decision to use adjuvant therapy. Examination of too few lymph nodes risks under staging a patient's disease. Failure to identify nodal metastases that exist may deprive the patient of adjuvant therapy and misinform them of their prognosis. Inaccurate staging may also have an adverse effect on survival statistics for both node negative and node positive cases. LN harvest is increasingly being suggested as a surrogate marker of surgical quality in the treatment of CRC [1, 2]. National agencies and professional associations in the UK and USA have recommended that a minimum of 12 nodes/patient should be examined, with all units being expected to achieve this level consistently [3–6].

LN retrieval is dependent on variables that relate to patient characteristics, the operation, and the techniques of both the operating surgeon and reporting pathologist [7–11]. There is also interunit variability in the harvesting of LNs following CRC resection [7, 12, 13]. It is not clear, however, whether the interunit variability previously observed is due to variations in patient characteristics, surgical technique, or pathological technique. The aim of this study was therefore to compare the LN harvest and factors influencing it in patients undergoing CRC resection by a single surgeon, in separate units, following relocation of the surgeon during the series.

## 2. Patients and Methods

The study population consisted of 213 patients undergoing consecutive potentially curative CRC resection for adenocarcinoma, operated on by a single consultant surgeon, in two units, over a seven-year period. In unit one 110 cases were carried out between October 2002 and July 2005 and 103 cases in unit two between August 2005 and October 2009. Patients were identified from prospectively collected databases at the two centres. Individual pathology reports were retrieved from the hospital pathology database and reviewed. All cases were carried out by an open technique, and there was no change in surgical technique during the study period. All cases were either performed by the consultant surgeon or by a trainee under direct supervision of the surgeon. CRC screening was introduced into the second unit during the study period, and seven cases performed in this unit were screen detected.

Pathological reporting of the resected specimens was performed by one of eleven consultant pathologists at the two units (three at unit one and eight at unit two). At the second unit, five pathologists had reported more than five specimens and the remaining three pathologists had reported less than five cases each. The results of the three pathologists reporting less than five cases were therefore pooled, totalling eight cases for analysis in this study. 

Both units had broadly similar pathological laboratory standard operating policies for the retrieval of LNs from CRC specimens which consisted of fixation in formalin, cutting through the mesenteric tissue in slices parallel to the bowel wall, followed by careful manual dissection of all LNs out of the specimen. Neither unit used fat clearing techniques. 

Data recorded for each patient and compared between units included overall LN harvest and case mix assessed by comparison of patient age, site of operation (divided into right colon, left colon, and rectum), operative urgency (elective or emergency), T stage (rectal cases treated with preoperative radiotherapy were excluded in analysis of this variable), and the use of neoadjuvant radiotherapy in rectal cancer.

Factors which may have influenced LN harvest (shown in Table 3) in addition to unit of operation were examined with univariate analysis. Significant factors on univariate examination were then assessed with multivariate analysis. Lymph node harvests, according to tumour location in right colon, left colon, and rectum, were recorded and compared between units.

The proportion of LN positive (Dukes' C) cases was compared between units and the LN harvest of LN positive and LN negative cases compared within the individual units. The effect overall LN harvest had on rates of LN positive cases across the whole series was also examined.

### 2.1. Statistical Analysis

Median values were used to compare all variables. Overall LN harvest between centres was compared using the Mann-Whitney *U* test. Case mix between the units was compared with Mann-Whitney *U* test and Chi-squared test, as appropriate. 

Factors influencing LN retrieval were examined with Pearson's correlation coefficient, Mann-Whitney *U* test, and Kruskal Wallis *H*-test as appropriate. The independent effect of variables that were significant on univariate analysis was assessed using multiple backward regression analysis. Significance was assumed for all tests at the 5% level. The data were analysed using SPSS version 16.0 for Mac statistical software (SPSS, Chicago, IL, USA).

## 3. Results

**Figure 1:**
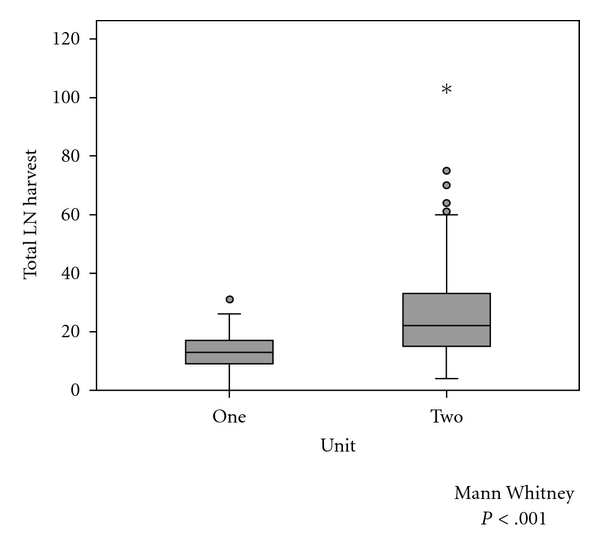
Boxplots of LN harvest at the two units. Grey boxes represent the interquartile range, black horizontal line within the grey box the median LN harvest, the and whiskers the range with circles representing statistical outliers.

There were 110 cases carried out in unit one and 103 cases in unit two. Overall median LN harvest was significantly different between units, in unit one 13 nodes/patient (range 0–30, 95% C.I 11.7–14.0) and in unit two 22 nodes/patient (range 4–102, 95% C.I 23.0–29.6), *P* < .001 (see Figure 1). Comparison of case mix, patient age, operative urgency, and tumour T stage is presented in Table 1. Case mix according to tumour site was similar between units (Table 1). 

### 3.1. Comparison of LN Yield according to Colonic or Rectal Tumour Location

Analysis of LN harvest according to whether the tumour was colonic (right and left combined) or rectal demonstrated that colonic (unit one median 15 nodes versus unit 2 median 18 nodes, *P* = .014) and rectal (unit one median 10 nodes versus unit two median 31 nodes, *P* < .001) tumours were higher in the second unit. Analysis of LN harvest according to tumour location demonstrated that LN harvests were significantly higher in left colonic and rectal tumours in the second unit, but identical in tumours of the right colon (Table 2). Intraunit analysis demonstrated that unit one had higher LN harvests in colonic cases (colon median 15 nodes versus rectum median 10 nodes, *P* < .001), whereas, in unit two, higher LN harvests were observed in rectal cases (colon median 18 nodes versus rectum 31 nodes, *P* ≤ .001).

### 3.2. Factors Influencing LN Retrieval

Speculative univariate analysis of the factors that may have influenced overall LN harvest, at the two centres, demonstrated that, in addition to the unit, significant variables for LN retrieval were T stage and reporting pathologist (Table 3). Age was not found to be a significant variable (Pearson's coefficient *r* = −0.048, *P* = .487), Backward linear regression analysis showed that unit (*P* < .001) and reporting pathologist (*P* = .007) were independent significant variables.

### 3.3. Proportion of Cases That Were Dukes' C according to Unit

In unit one 46/110 (42%) cases were LN positive and in unit two 49/103 (48%), *x* ^2^ *P* = .398. In unit one, the median LN harvest of patients who were LN negative was 11 nodes/patient and in those who were LN positive was 15 nodes/patient, *P* = .004. In unit two the median LN harvest in patients who were node negative was 21 nodes/patient and in those who were node positive was 23 nodes/patient, *P* = .616.

### 3.4. Effect of LN Harvest on Identification of LN Metastases

**Figure 2:**
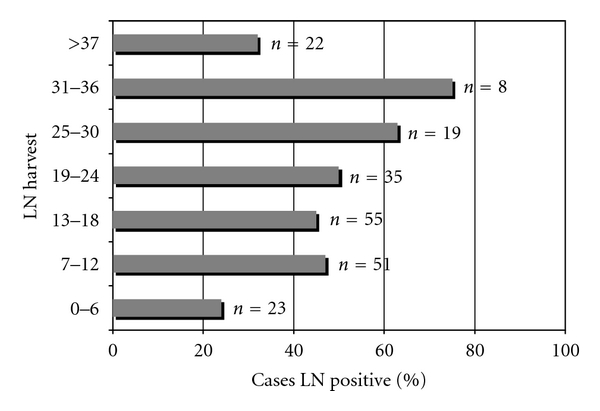
Lymph node harvest and percentage of cases of lymph node positive.

The effect of LN harvest on the identification of LN metastases is presented in Figure 2. Increased frequency of finding at least one metastatic node (Dukes' C) was seen up to a harvest level of 36 nodes/patient.

## 4. Discussion

Accurate histopathological staging of colorectal cancer (CRC) is vital to identify patients with Dukes' C disease for adjuvant chemotherapy. In addition, accurate staging is imperative to provide patients with realistic prognostic information and to allow meaningful comparative audit between units. This is particularly important as lymph node (LN) harvests are increasingly being used as surrogate markers of surgical quality in the treatment of bowel cancer [1, 2]. Previous studies have demonstrated that LN harvests are dependent on numerous variables that relate to patient characteristics and the techniques of surgeon and pathologist [7–11].

The present study has demonstrated that a surgeon relocating to a new unit may experience a dramatic and statistically significant increase in nodal yield following resection for colon and rectal cancer, despite no change in surgical technique, and with a similar case mix in terms of patient age, tumour location, and T stage. The implication of this finding is that the difference in LN retrieval between units relates to the pathological techniques, as the surgical technique has been standardised by the surgeon. 

Review of the standard operating policies of both laboratories showed no discernable difference in the methods of fixation or specimen dissection, which suggests that the differences must relate to the individual pathologist. Neither laboratory employed fat clearance techniques that have previously been reported to increase both nodal yields and upstage tumours [14–17]. Fat clearance techniques have not routinely been used in most centres, and we believe the methods used in this study are representative of practice across the UK.

It has previously been reported that LN harvests following rectal resection are lower than after colonic resection [7, 8, 13, 18]. This may explain some of the lower LN harvest observed in unit one in this series, where proportionally more rectal resections were performed. However, in unit two, rectal cancer specimens had significantly higher LN yields than colonic tumours. The use of preoperative radiotherapy for rectal cancer in unit two was not associated with a reduced lymphatic harvest, as has been previously widely reported [7, 8, 19, 20]. A possible explanation, for these apparent divergences from the norm, is that a pathologist with a particular interest in rectal cancer specimens reported most of the rectal cases in unit two.

Another possible explanation for the observed difference in LN harvest between unit one and two is the separate chronological time periods that the harvests cover, that is, unit one years 2002–2005 and unit two years 2005–2009. During the latter period, national nodal harvests across the UK have improved [21]. However, local audit of LN harvesting at unit one, for resections between 1999 and 2004, showed a median of 13 nodes/patient [8]. Reaudit of harvests at unit one for the period 2006-2007 showed that the median harvest was identical, at 13 nodes/patient [22].

Higher LN harvests are associated with higher rates of both node positive disease and improved survival [7, 8, 12, 23–26]. In the present study, increased nodal yield at the second unit was associated with a trend towards a higher proportion of cases being staged as Dukes' C. Although the difference was not statistically significant, it is possible that this represents a type II statistical error and that a larger data set may yield a statistically significant result. 

Whether 12 nodes per patient is an appropriate level is controversial. It may be appropriate that the guidance should be revised so that as many nodes as possible should be examined [7, 23, 27]. This study supports the latter view with a higher proportion of cases being classified as LN positive cases up to 36 nodes/patient. Our findings must, however, be interpreted with some caution as more pathologically advanced tumours have been associated with higher LN harvests [13]. The higher nodal harvest observed may therefore be a consequence of the disease severity, rather than patients with lower LN yields having missed nodal metastases. 

In this series, following multivariate analysis, unit of operation and reporting pathologist were independently predictive of nodal harvest. Our finding that a surgeon working at different sites can experience different nodal yields has been previously reported (Rieger et al. [28]), although this series was smaller than our study and the results were not subjected to multi-variate analysis to determine if pathologist or unit were independently predictive of LN harvest.

## 5. Conclusions

This study has demonstrated that a single surgeon who moves units, with no change in surgical technique and similar case mix at the two units, can experience significantly differing LN harvests following resection for CRC. These findings confirm that the pathologist is a critical determinant on the numbers of LNs harvested following resection for CRC. This has implications if LN harvest is used as a marker of “surgical quality.”

## Figures and Tables

**Table 1:** Case mix between units.

		Unit 1	Unit 2	*X* ^2^
		Percentage of total cases	Percentage of total cases	*P* value
Tumour location	Right colon*	35%	40%	*P* = .427
		(38/110)	(41/103)	
	Left colon**	20%	25%	*P* = .360
		(22/110)	(26/103)	
	Rectum	45%	35%	*P* = .153
		(49/110)	(36/103)	
	Panproctocolectomy	1%	0%	NA
		(1/110)		

Median patient age		72	71	*P* = .789

Operative urgency	Elective	86%	90%	*P* = .373
		(95/110)	(94/103)	
	Emergency	14%	10%	
		(15/110)	(10/103)	

T stage***	1 & 2	21%	20%	*P* = .857
		(19/89)	(17/84)	
	3 & 4	79%	79%	
		(71/89)	(67/84)	

*Right colon includes right hemicolectomy, extended right hemicolectomy, subtotal colectomy, and transverse colectomy. **Left colon includes left hemicolectomy, sigmoid colectomy, and Hartmann's procedure for colonic tumours and high anterior resection for colonic/rectosigmoid tumours. ***Rectums with preoperative radiotherapy excluded.

**Table 2:** Lymph node harvest according to tumour location between units.

	Unit 1	Unit 2	*P* value
	Median LN harvest/patient	Median LN harvest/patient	
	(range)	(range)	
Right colon	16 (5–26)	17 (5–47)	.253

Left colon	15 (6–30)	21 (4–64)	.023

Rectum (overall)	10 (0–22)	31 (5–102)	<.001

Rectum without preoperative radiotherapy	11 (0–22)	25 (5–102)	<.001
	*n* = 28	*n* = 17	

Rectum with preoperative radiotherapy	7 (1–20)	41 (20–70)	<.001
	*n* = 21	*n* = 19	

**Table 3:** Analysis of factors that may have influenced overall LN retrieval.

Variable			Number	Median LN harvest	*P* value
Unit		Unit 1	110	13	*P* < .001*
		Unit 2	103	22	

Operation type		Right colon	80	16	*P* = .761**
		Left colon	48	17	
		Rectal	85	16	
		Rectal with radiotherapy	40	16	*P* = .996*
		Rectal without radiotherapy	45	19	

Operative urgency		Elective	188	16	*P* = .299*
		Emergency	25	15	

Final Dukes' stage		A	45	12	*P* = .158**
		B	72	16	
		C	96	17	

T stage		CR	7	7	*P* = .001**
		1	14	9	
		2	40	18	
		3	114	16	
		4	38	17	

Reporting Pathologist	Unit 1	1	31	15	*P* < .001**
		2	39	14	
		3	40	11	

Reporting Pathologist	Unit 2	4	37	33	*P* < .001**
		5	32	15	
		6	12	19	
		7	8	23	
		8	6	25	
		9***	8	24	

Clinical presentation		Symptomatic	205	16	*P* = .195
		Screen detected (all unit 2)	8	19	

*Mann-Whitney *U* test. **Kruskal- Wallis *H* test. ***Pooled results of 4 pathologists each reporting less than 5 cases.
